# Frontline Healthcare Workers’ Reflections on Operationalizing the COVID‐19 Pandemic Response: A Qualitative Study

**DOI:** 10.1155/jonm/8599692

**Published:** 2026-07-08

**Authors:** Karen McKenna, Stéphane Bouchoucha, Bernice Redley, Ana Hutchinson

**Affiliations:** ^1^ Australasian College for Infection Prevention and Control, Hobart, Australia; ^2^ School of Nursing and Midwifery, Deakin University, Geelong, Victoria, Australia, deakin.edu.au; ^3^ School of Nursing and Midwifery, Centre for Quality and Patient Safety Research in the Institute for Health Transformation, Deakin University, Geelong, Victoria, Australia, deakin.edu.au; ^4^ Centre for Innovation in Infectious Disease and Immunology Research (CIIDIR), Deakin University, Geelong, Victoria, Australia, deakin.edu.au; ^5^ Manipal Academy of Higher Education (MAHE), Manipal, India, manipal.edu; ^6^ Centre for Quality and Patient Safety Research in the Institute for Health Transformation, School of Nursing and Midwifery, Deakin University Geelong, Geelong, Victoria, Australia, deakin.edu.au; ^7^ School of Nursing and Midwifery, Centre for Quality and Patient Safety Research in the Institute for Health Transformation, Epworth Health/Deakin University Partnership, Geelong, Victoria, Australia, deakin.edu.au

**Keywords:** clinical leadership, COVID-19 pandemic, crisis communication, frontline healthcare workers, operationalization of pandemic plans

## Abstract

**Introduction:**

The COVID‐19 pandemic provided opportunities to learn from healthcare workers involved in implementing pandemic plans within healthcare settings. This study explores healthcare workers’ reflections on the first 3 years of the COVID‐19 pandemic and their experiences activating health service pandemic plans.

**Methods:**

A qualitative exploratory descriptive study design was used to explore through focus groups’ frontline healthcare workers’ experiences related to emergency response planning, operationalizing the pandemic response plan, the workforce mindset, and re‐evaluation of the organizations’ response once the crisis had abated. Thematic analysis guided by Braun and Clark’s six‐steps was used to identify themes.

**Results:**

Thirty‐two healthcare workers participated in 10 focus group discussions. The following six key themes were identified and explored (1): unprepared emergency response systems and processes, (2) adaptive crisis management under pressure, (3) the evolving reality of the pandemic response and embedding the basics, (4) leadership styles and communication challenges when managing a crisis, (5) psychological challenges of leading the workforce during a prolonged crisis, and (6) organizational preparedness for future pandemics.

**Conclusion:**

Healthcare workers were required to respond rapidly to maintain safety and meet healthcare needs, with little understanding of the organization’s pandemic plan. This study provides an opportunity to learn from the experiences of healthcare workers in integrating the frontline perspective into the design and execution of emergency response plans, to enhance workforce sustainability and organizational preparedness.

**Practitioner Points**
• Frontline healthcare workers (HCWs) need regular simulation exercises to develop skills in implementing emergency response protocols.
• Simulated emergency response training may provide benefits and empower frontline HCWs.
• Development of a structural organizational communication strategy is needed from the onset of a pandemic, to support HCW well‐being and improve operational efficiency and effectiveness.

## 1. Introduction

The COVID‐19 pandemic provides a unique opportunity to explore and learn from the experiences of front‐line healthcare workers (HCWs) and clinical leaders in implementing emergency and pandemic response plans, and the impact this had on health service resilience, including service provision and frontline HCW well‐being [1]. The initial impact of the pandemic on healthcare services before and during the first COVID‐19 waves was profound, requiring rapid changes to modes of service delivery [2]. Adapting and changing organizational processes to provide essential services, manage the workforce, and sustain the pandemic response over several years was also required. Although numerous studies have documented frontline workers’ experiences during the COVID‐19 pandemic [3–5], there is limited research capturing clinicians’ insights on how to improve preparation and operationalization of a pandemic response over the 3 years of heightened activity managing COVID‐19 infections. There is now an opportunity to reflect on the initial and sustained impact of the pandemic on healthcare settings and to use these learnings to address healthcare system resilience and vulnerabilities, and to shape system‐wide performance, emergency planning, and preparedness [6].

During the first wave of the pandemic (February–June 2020), Australian borders were closed to all nonresident travelers, and a mandatory 14‐day hotel quarantine for international arrivals was implemented, combined with a national lockdown that included stay‐at‐home orders, online schooling, and businesses closing. Community transmission rates were minimized with most COVID‐19 cases associated with international arrivals [7]. Measures to suppress community transmission attempted to protect the health system, which relied on healthcare providers implementing isolation restrictions and lockdown protocols to suppress transmission [8, 9]. Although this swift Australian response initially shielded the population from intense and severe outbreaks, by mid‐2020, there was a second wave of community transmission in Melbourne, Victoria. The substantial wave occurred from late June to August 2020, with Victorian case numbers peaking at around 500–700 cases each day [10, 11], resulting in surges in acute care demand [7]. Strict restrictions limiting travel, curtailing social contact, and introducing curfews saw the case numbers decrease by September 2020 to around 30–50 cases per day [10]. One local government area in greater Melbourne (The city of Wyndham) recorded the highest number of cases during this time, with 2248 cases in a population of 270,487 residents [12].

The COVID‐19 pandemic disrupted health service delivery, and frontline healthcare providers faced immense challenges executing a pandemic response that tested resilience and required the redesign of systems and processes in real time [13–15]. At the onset of the COVID‐19 pandemic, before vaccines became available, HCWs experienced heightened emotions, anxiety, fatigue, and fear, while continuing to provide care to patients and their communities [16–18]. During this time, access to scientific knowledge was limited, and there was heavy reliance on media updates for information [19]. In addition, Australian HCWs were exposed to intense international media coverage of large‐scale outbreaks and deaths internationally, which further amplified HCWs’ level of emotional distress [19].

These challenges highlighted the importance of understanding the preparedness, adaptability, and resilience of health services during periods of normal activity and in the event of emergency situations. Health system resilience is defined as the ability of the health system to anticipate, prepare for, and adapt to shocks or stressors, while continuing to deliver care to the population [20]. Health system resilience frameworks conceptualize this and provide tools to assess the preparedness of the health system [21]. The health system resilience analytical framework by Reiss, Kraus [21] comprises two dimensions that distinguish between normal prerequisites, which are established during normal operational times, and response strategies implemented in the event of a crisis [21]. The analytical framework was designed using six domains that can be applied as a tool to assess the resilience of a health system and includes governance and leadership, information and communication systems, financing, and physical and human resources [21].

To date, much research has focused on the impact of the pandemic on frontline HCWs in terms of psychological well‐being [22–24]. There is limited evidence that examines the experiences of frontline HCWs in activating and implementing a pandemic response during the COVID‐19 pandemic, through the lens of health system resilience. By focusing on these aspects, we can gain a multilevel perspective on the challenges faced during the pandemic and how these challenges evolved over the course of the pandemic [15]. Understanding how the health service governance structures, communication systems, and workforce support performed within the clinical setting during the pandemic is essential to strengthen the health services’ operational capabilities during a time of crisis, and to better prepare the organization for future emergency events. The aims in this study were to: explore the perspectives of frontline HCWs in implementing the organizational response to the COVID‐19 pandemic using the analytical resilience framework and to inform improvements in organizational emergency and pandemic response planning. This study offers a unique perspective as it captures frontline HCWs’ experiences looking back over the first 3 years of the pandemic response.

## 2. Methods

The setting for this research is a tertiary health service provider with a network of public hospitals in Melbourne, Victoria, providing care to the population across the public sector. The health service includes primary, subacute ambulatory care, and acute care delivery. This health service was selected as it is a medium‐sized service with multiple, geographically dispersed sites that provide a comprehensive range of acute care services, enabling exploration of the organization’s preparedness and response to the pandemic across different operational contexts.

Two acute care sites within the health service network were included in the study. Site A provides specialist maternal and child health services, and site B provides general acute care services that include an emergency department, critical care, as well as general medical and surgical inpatient units. During the first 3 years of the pandemic, site A experienced lower‐case numbers; however, they were required to undertake rapid changes to service delivery models to continue to provide essential obstetric care during the pandemic [14, 25]. In contrast, site B experienced a substantial impact largely based on its geographic location in an epicenter for large‐scale COVID‐19 outbreaks [12].

### 2.1. Study Design

A qualitative exploratory descriptive (QED) study involving focus group discussions with frontline HCWs was conducted between June and December 2023 [26, 27]. Focus groups were chosen to allow participants to interact and share experiences and to provide depth to their responses through interactive discussions with colleagues.

### 2.2. Study Participants

To obtain a broad range of perspectives, participants were recruited from a wide range of clinical specialties, departments, and services. Participants included HCWs working in general wards, critical care areas (emergency department and intensive care units), and specialty COVID wards, as well as those in frontline clinical leadership roles. Inclusion criteria were: HCWs who worked in clinical areas during the pandemic and experienced the impact of the organization’s emergency response plan and provided consent for study participation. Exclusion criteria were: HCWs who were not employed at the health service in 2020 and therefore not involved in the implementation of the pandemic response and staff who were not permanent employees.

### 2.3. Recruitment

Purposive sampling was used to obtain a representative sample of HCWs who worked in clinical areas that were impacted during the pandemic and had knowledge and experience of the health service pandemic response. Study information was distributed to potential participants via department‐level staff email groups, and interested individuals responded directly to the research team. Further information was provided about the research project, and eligibility confirmed via email. The email invite was distributed to 92 participants, with 32 agreeing to participate. The remaining 60 participants either declined or did not respond. Informed consent was obtained prior to the focus groups commencing.

### 2.4. Data Collection

Focus groups were made up of HCWs working in similar roles and levels of seniority. Grouping participants with similar roles and level of seniority was intended to facilitate an open discussion, where participants felt comfortable to express their views, challenge organizational processes, and share their reflections, enhancing both the depth and quality of the data. When preexisting relationships between one researcher who was employed in the health service (Karen McKenna) and participants were evident, another researcher (Ana Hutchinson), independent of the health service, conducted the focus group. All participants’ information was coded to ensure anonymity, and no participants dropped out of the study.

The focus groups were conducted over the Zoom online video platform with two researchers present (Karen McKenna and Ana Hutchinson) to facilitate the discussion. A third researcher (Stéphane Bouchoucha) reviewed the thematic analysis independently to confirm that the reported findings were representative of the focus group transcripts. All researchers had experience and expertise in qualitative research methods and infection prevention and control practices in healthcare settings. Reflective practices were undertaken throughout the study, including regular discussions and reflection during data collection and analysis. Member checking was offered to all participants but was not taken up. Credibility was supported through reflexive discussion, analysis, and alignment of the data and findings. Data saturation was achieved when no new themes emerged, and thematic patterns were established. This study was reported in accordance with the consolidated criteria for reporting qualitative research (COREQ) checklist.

Focus group discussions lasted between 33 and 72 min and were recorded for later transcription. The focus group interview guide was developed to explore frontline workers’ experiences operationalizing the organization’s pandemic response plan over the first three years of the COVID‐19 pandemic. The topics explored were: (1) emergency response planning, (2) experience operationalizing the pandemic response, (3) workforce mindset, and (4) reevaluation and recommendations for the future.

### 2.5. Data Analysis

The audio file of the video recordings of the focus groups and field notes were transcribed by one of the researchers (Karen McKenna) and checked against the recordings for accuracy. Transcripts were uploaded into NVivo software, which was used as a data repository to keep a systematic record of each step in the analysis. A deductive thematic analysis was undertaken which was guided by Braun and Clarke’s [28] six steps, including developing familiarity with the data, systematically generating codes and grouping codes through all transcripts, refocusing the analysis into broad themes, and a two‐level review and refinement of themes. This was followed by defining and naming the themes and analyzing the data. Coding and identification of themes were done by researcher Karen McKenna and then reviewed by a second researcher (Ana Hutchinson). Subthemes were mapped to the following phases of the pandemic response— preparation, operationalization, post‐pandemic recovery, and the analytical resilience framework domains. The responses were collated based on the site of the participant to determine potential differences in the pandemic response between different sites. A third researcher (Stéphane Bouchoucha) reviewed the thematic analysis independently to confirm that the reported findings were representative of the focus group transcripts.

The authors declare that no artificial intelligence–generated content tools were used in the design, data collection, analysis, or preparation of this manuscript.

### 2.6. Ethical Considerations

Ethics approvals were obtained from the institutional Human Research Ethics Committee and the University.

## 3. Findings

A total of 32 staff participated in 10 focus group discussions between June and December 2023. Focus groups included 1–8 participants per group and lasted from 33 to 72 min (Table 1). Five participants were male, and 27 were female. Twenty participants were from the general acute hospital (site B), 10 from the specialist acute hospital (site A), with 2 staff who worked across both sites. Clinical roles included registered nurses/midwives (*n* = 30) and medical doctors (*n* = 2). Within the registered nurse/midwife group, roles included nurse unit managers (NUMs) (*n* = 10), associate nurse unit managers (ANUMs) (*n* = 3), clinical educators (*n* = 2), registered nurses (*n* = 8), midwives (*n* = 2), and infection prevention nurse consultants (*n* = 5).

**TABLE 1:** Focus group composition.

Interview number	Duration (min)	Participants (number)	Site	Role
1	45	2	B	ANUM—ED, OT
2	33	3	B	RN—COVID‐19 wards
3	27	1	B	ANUM—ICU
4	55	8	A/B	RNs—ED, Med ward, DOSA, mental health
5	49	3	A	NUMs—ED, BSU, COVID‐19 ward
6	58	3	A	NUMs—ED, patient access, outpatients
7	72	2	B	RNs—NICU
8	71	2	B	NUM & doctor—ICU
9	47	3	B	NUMs—OT, ED, COVID‐19 ward
10	64	5	A/B	RNs—IPC

*Note:* Site A—Specialist maternal and child health services. Site B—General, acute, subacute, and mental health service.

Abbreviations: ANUM, associate nurse unit manager; BSU, birth suite unit; DOSA, day of surgery admissions; ED, emergency department; ICU, intensive care unit; IPC, infection prevention and control; NICU, neonatal intensive care unit; NUMs, nurse unit managers; OT, operating theaters; RN, registered nurse.

The themes and subthemes identified in relation to each discussion topic are shown in Table 2.

**TABLE 2:** Themes and subthemes identified.

Topic	Theme	Domain	Subtheme
1. Emergency response planning	Unprepared emergency response systems and processes	• Governance and leadership • Service delivery • Physical resources	i. Watching and learning from the international experience
			ii. The organizational response was chaotic and underresourced
			iii. Lack of a command‐and‐control approach
			iv. Frontline staff lacked emergency response training and were inadequately prepared

2. Experience operationalizing the pandemic response	Adaptive crisis management under pressure	• Physical resources • Human resources • Service delivery	i. Tightly balancing priorities between maintaining essential services and ensuring adequate staffing
			ii. Feeling vulnerable when making decisions on the fly
			iii. The impact on patients and visitors could be devastating

3. Workforce mindset	Crisis leadership and supporting a workforce under pressure	• Service delivery • Human resources • Information and research	i. Fear and distrust in the pandemic response
			ii. Emotional and physical exhaustion among HCWs
			iii. Learning to adapt and cope with constant change
			iv. The impossible task of communicating to the frontline.

4. Reevaluation and recommendations for the future	Emergency leadership and planning for future pandemics	• Governance and leadership	i. Developing an updated pandemic response plan that is fit for purpose

### 3.1. Theme ‐1: Unprepared Emergency Response Systems and Processes

The first theme indicated that participants thought that the health service lacked critical resilience prerequisites relating to governance, leadership, and service delivery domains and demonstrated “Unprepared emergency response systems and processes” in the early stages of the pandemic. Frontline HCWs lacked awareness of leadership roles and responsibilities that led to confusion and inefficiencies in the coordination of the pandemic response. Subthemes identified were (1.1) watching and learning from the international experience, (1.2) the organizational response was chaotic and underresourced, (1.3) lack of a command‐and‐control approach, and (1.4) frontline staff lacked emergency response training and were inadequately prepared.

#### 3.1.1. Watching and Learning From the International Experience

Participants observed that the health service was in a fortunate position, as they were able to watch and learn from international events early in 2020. At site B, participants described taking a proactive approach and being able to prepare their clinical settings to care for patients with COVID‐19 infections, “I also feel that watching Europe gave us a bit of time to be prepared, we were able to do simulated intubations, and transports. We prepared some of that stuff prior and practiced as well” (P24). In contrast, variations in how early warning opportunities were operationalized across the two sites were evident, with participants from site A indicating that there was some complacency with little action from health service leaders to prepare for potential cases during this time. “We could see the storm across the mountains, and it was coming down on us, and we were all standing there waiting for someone to do something” (P15).

#### 3.1.2. The Organizational Response Was Chaotic and Underresourced

Multiple participants from both sites observed that the initial response from the organization was chaotic, unprepared, and they were lacking the resources needed to implement a response, describing it as fragmented and reactive, with resource limitations that undermined the health services’ ability to respond effectively.

We had this mad rush to try and prepare and redesign our entire service to manage it, which was a lot of logistics that were involved, and nobody knew what they were doing. And it was just a bit of a mess. (P15 Site A)

We were just really not prepared when it comes to resources, staff probably, especially in infection control, there was just not enough staff for them to deal with everything [and]…There was a lot of unpreparedness, a lot of gaps I feel in communication and resources. (P17 Site A)

Two respondents felt that the organizational leaders were aware of those gaps and that they were able to be closed relatively quickly, “I would say initially there were gaps, but we did close those gaps pretty quickly” (P17 Site A) and “I think some of it, some of the readiness and agility and how to do things and make changes, and how we looked after patients, I think some of that all went really well” (P24 Site B).

#### 3.1.3. Lack of a Command‐and‐Control Approach

The participants described a lack of central coordinated response by the health service governance and leadership structure, commenting, “I feel like there was a huge disconnect, I don’t know what was happening from a health services, organization, incident command level response, but that seemed to be happening on another planet compared with the planet we were on” (P19 Site A) and “It took a long time to get it together, to have that one governing body to manage, which should have probably happened from the start” (P15 Site A). Instead, participants reported that the lack of a centralized command and control response left frontline leaders to initiate a local response, “In that early stage there wasn’t a lot of support for change or for implementation of strategies, so it was very much of just step up and act, without direction” (P19 Site A). This lack of central coordination increased HCWs anxieties in the early stages of the pandemic.

I think those constant changes also made staff feel that the leadership team and management don’t know what they’re doing […]. It took a long time until we made some plans, and we were all on the same page, just with our one hospital. [Before that], literally everyone was doing their own thing and the staff were just not happy. (P17 Site A)

Participants from both sites observed a disconnect between the executive leadership response and the reality that was faced on the frontline, “I think it exposed a disconnect between decision makers and the clinical staff at the coal face” (P23 Site B). Although they acknowledged that the pandemic was an unknown quantity and the leadership team was also learning as they went,

We didn’t fully recognize the length of time this was going to be around for, and the full impact of it, and I think that is why the communication wasn’t as clear as it could or should have been, and I think that is because we were all in the dark and we were all learning as we went, the executive as well. (P20 Site A)

Participants also reported that trying to fit policies and procedures into practice that were not designed for the local practice context was cumbersome.

I do think the governance, how the Health Service ran the governance particularly was a problem, because it was over the whole of health services and aged care, and it meant that we weren’t being able to have decisions that make sense in our area because we had to fit in this clunky structure that was also being organized for aged care. (P15 Site A)

#### 3.1.4. Frontline Staff Lacked Emergency Response Training and Were Inadequately Prepared

Emergency response and disaster management training was identified as lacking and found to be a gap in workforce preparedness and resilience of the health service, which impacted the overall effectiveness of the emergency response.

I think our hospital [Site B] specifically, rapidly we came to realize that we did not have the depth of staffing, the breadth of clinical skill, the availability of the necessary specialties, and the availability of the required equipment, we had been writing coincidentally around that time, business cases for buying the equipment that the department of health suddenly gave to us. (P23 Site B)

I don’t think disaster management and disaster planning is usually built into organizational culture, and I’m thinking back to my days as a pre‐vocational registrar, over 20 years ago now, not only were we aware that our senior medical staff were actively involved at a hospital and a health service level, and with their local departments of health in disaster management and planning, and they were the ones who were actively involved. (P23 Site B)

### 3.2. Theme 2: Adaptive Crisis Management Under Pressure

The second theme explored “Adaptive crisis management under pressure” and the emergency response strategies that were implemented in response to the pandemic. Hospitals faced unprecedented challenges during the pandemic that required adaptive, flexible, and rapid decision‐making capabilities to address pandemic challenges while maintaining essential service delivery. Site B was located in a COVID‐19 epicenter and experienced surges in COVID‐19 patient admissions, while site A faced changes to delivery of essential services such as provision of maternity care. Subthemes identified within this theme were (2.1) Tightly balancing priorities and (2.2) Feeling vulnerable when making decisions on the fly.

#### 3.2.1. Tightly Balancing Priorities

The impact of maintaining essential services was explored by site B participants, with two participants reporting that the health service implemented adaptive decision‐making within the service delivery and workforce domains, through reducing elective surgeries to focus on emergency category 1 and 2 cases. This adaptive response benefited the health service by reducing the number of COVID‐19 positive patients entering the facility, and the reduced surgical case numbers meant the operating theaters (operating rooms) were adequately staffed when staff furlough numbers increased.

We reduced the elective theater workload…. We closed theaters, we stopped doing elective cases, and when there were bad outbreaks, we cut down on doing Cat [egory] 3 and Cat 2’s to reduce the risk of having positive patients coming through. We were continuing Cat 1’s [urgent surgical cases] in a lot of those lockdowns. Obviously we had to keep doing C‐sections, and we were doing cancers and things like that, but people with a hemorrhoid could wait. (P2 Site A)

#### 3.2.2. Feeling Vulnerable When Making Decisions on the Fly

Multiple participants across both sites reported a sense of vulnerability from the lack of organization and preparedness in the initial stages of the pandemic, and the lack of a pandemic blueprint and leadership direction resulted in the response strategies being implemented locally by area managers, reflecting a decentralized leadership response.

By the first wave, the other organizations were more organized than us, we were looking at what they were doing rather than taking our own steps during the first one. We were definitely reacting rather than being proactive. (P6 Site B)

In the early stage there wasn’t a lot of support for change or for implementation of strategies, so it was very much just step up and act, without direction, so we did. (P20 site A)

So I was just listening to the ED manager say, oh I was doing this and another manager was doing this. And on level 5 and we had 3 managers and we kind of worked together, but it was a bit disappointing that initially it took every ward, doing their own stuff, which should have probably been managed altogether. (P17 Site A)

The response from site A participants indicated a greater sense of vulnerability, and a lack of preparedness, which could be explained by the specialist nature of the service and less frequent exposure to emergency responses, “We didn’t know what we needed to do, and the things that we could figure out that we needed to do, we had to figure out how to bring them in, but with no help” (P19 Site A).

### 3.3. Theme 3: Crisis Leadership and Supporting a Workforce Under Pressure

The third theme explored crisis leadership and supporting a workforce under pressure. Participants reflected on the challenges faced within the domains of service delivery, information and communication, and human resources and well‐being. Four subthemes were identified: (3.1) Fear and distrust in the pandemic response, (3.2) Emotional and physical exhaustion among HCWs, (3.3) Learning to adapt and cope with constant change, and (3.4) The impossible task of communicating to the frontline.

#### 3.3.1. Fear and Distrust in the Pandemic Response

Though site B faced significant challenges with increased cases of COVID‐19 impacting clinical care, the participants from both sites reported that fear was a significant factor impacting the workforce, and that dealing with staff emotions was challenging.

Everyone’s emotions, angry, crying, and I think those constant changes made staff feel that the leadership team and management, they don’t know what they’re doing.” (P17 Site A)

And everybody’s primary concern was obviously themselves and their families, and trying to manage the whole emotional side of people’s responses to things as well was an incredible amount of work.” (P19 Site A)

Distrust in the organizational leadership compounding this issue, for example “The staff they were looking at the news and what’s happening overseas and then coming to us like what are we doing, why are we not doing it, because they couldn’t see on the ground level what was happening” (P6 Site B).

Fear of exposure to COVID‐19 and potential impacts on family members’ health was a primary concern throughout the pandemic. “I think there’s a lot of fear, people who we notified as close contacts were very fearful that they’d get COVID‐19 and die potentially. I think staff were very, very scared, and so were family members” (P30, Site B). Participants also reported the traumatic impact that multiple exposures had on the workforce.

There’s a lot of very frightened staff who were absolutely traumatized because we had incident after incident, where we had exposures from patients to staff, and then the wait for 24 hours to see if they had COVID‐19. I had numerous staff crying with me, worried that they were going to kill their parents, their husbands… all that sort of stuff. (P15, Site B)

#### 3.3.2. Emotional and Physical Exhaustion Among Healthcare Workers

Participants described how the impact of exhaustion was not felt until there was a downturn in the cases and exposures, and everyone had an opportunity to reflect on the toll of the pandemic, “I suppose for our own well‐being there wasn’t the time to reflect about how we were feeling and in that downturn, and then before the second peak I suppose we had that time to reflect on how we were all feeling and how tired most of us were” (P31 Site B). One participant reported that fear impacted patient care, requiring strategies to educate and reinforce safe practices.

I think it threw me that people didn’t have a commitment or a loyalty to care for residents and patients, […] there’s no way that I could take that on board and just go well I’m not going to look after them! I was trying to convince people that if you use all your PPE and did it correctly, they were going to be okay”(P29 Site B)

#### 3.3.3. Learning to Adapt and Cope With Constant Change

Indirect impacts on team relationships were substantial, with participants reporting that disruptions resulted in a cycle of making and breaking relationships. “I think relationships were made or broken during that time, but it was just a constant continuous cycle of breaking a relationship, re‐doing it, breaking it, re‐doing it, which is really difficult for the managers” (P16, Site A). In addition, redeployment and movement of workforce to other departments impacted staff morale, “So it was hard for the re‐deployed staff I think, especially getting a full patient load when you have never done it before, so there was a lot of unhappy people, but I think a lot of people just saw that they needed to dig in as well and help out” (P1, Site B).

Despite these challenges, participants reported that ultimately frontline teams come together and helped each other through difficult times. For example, “I think in our organization we kind of just all banded together. That is one of the good things that came out of COVID. We all stepped up, we came together (P15, Site A); and “We did our own little fundraising for a welfare club, and we bought groceries and things for everybody, all our staff that were off sick, when they were not just the isolating but sick, so if you turn it round there were a lot of positives that bought us quite close that way” (P7, Site B).

#### 3.3.4. The Impossible Task of Communicating to the Frontline

Disseminating information that evolved rapidly to large groups of HCWs was challenging, and this resulted in local area managers developing their own team communication strategies. This lack of embedded and robust communication structures was identified as a barrier to effective information exchange.

I think generally in the organization this wasn’t something that had been thought about, so when it happened it felt like it was very challenging to get information quickly, and information changed very fast, so even within one day you might have been on one afternoon and come back the next morning and things had already changed and we had to be like ‘what is happening now?’ (P1 Site B)

I definitely remember being on the phone regularly, all the time, like feeding up constantly the issues and what should we do and that sort of thing […] the actual doing and the communication didn’t have direction…In those early days we didn’t have that leadership communication that was as clear as we would have liked. (P20 Site A)

This resulted in areas such as the ICU and anesthetic teams developing their own protocols based on guidance from experts outside the organization, for example,

I was frustrated with the communication you got in an email, because sometimes it was a little bit of contradiction, and who did you believe, because we had a lot of divisional directors and leaders here, so we had infection control, we had ICU, you had different divisions, so it was very difficult for us to map out a pathway in relation to who we follow, because ICU would run something differently. …But we had our rules in place, I felt that we followed them accordingly. (P25 Site B)

Making sure the anesthetic staff were being kept up to date, so of course they were working at other health services. And that health service was doing that and they would want to come and try to do the same thing here and we were saying No, you have to follow our rules, so it was difficult from that perspective. (P26 Site B)

Fast‐changing government‐initiated guideline updates contributed to communication challenges between leaders and frontline HCWs. The timing of updates at 5 pm on a Friday greatly impacted frontline staff, with several participants reporting having to implement these changes prior to the weekend with limited resources and support. “And then it came out on a Friday afternoon, and then you running around looking for the equipment, putting out notices, sending out memos, printing them off, making sure everybody knows” (P16, Site A). Ineffective communication also contributed to a lack of confidence in the organization’s capacity to respond to future pandemics or health emergencies, “I would say if a different pandemic showed up and it wasn’t a respiratory pandemic next time, we would potentially be in the same boat” (P23, Site B), “I am not confident that if similar things were to happen tomorrow, I am not confident as an organizational culture we would change that response” (P24, Site B).

### 3.4. Theme 4: Planning for Future Pandemics

The fourth theme was around the participants’ reflections on future‐proofing the health service, and the need to strengthen the organization’s resilience when preparing for future pandemics or emergency events. Within this theme, participants spoke about the value of emergency response planning in developing a pandemic plan that operated like a blueprint for each department, providing operational guidance and strengthening the domains of governance and leadership, service delivery, workforce preparedness, and resources.

One participant (from Site B) also identified the need for the lessons learned from the COVID‐19 pandemic to be given consideration when building new hospital infrastructure, while another identified that a lack of documentation and checklists to guide front‐line HCWs when implementing the plan in their clinical area was lacking.

I think anything like that (emergency response planning) is going to be incredibly valuable […] I think having any sort of disaster management planning and working out what will happen and what will go into play, and making sure that they are being run every year, so that it like a well‐oiled machine when it does happen. (P1, Site B)

I think that would be useful, what worked for that department at the end. And it doesn’t mean that they have to set in stone this is how you have to manage it, cause the disease might be different. (P2, Site B)

We should be really thinking about design a bit more, […] it’s probably something that should be taken into consideration, how would we manage if we needed to isolate and whether or not that’s a consideration. I would like to think it is, but I am not necessarily convinced that it would be.” (P1, Site B)

That’s a good point, we don’t even practise those things, on any given day we are going to have an increase from a train accident or respiratory, to an outbreak of something, so it is around how we handle that emergency planning, which I’m used to from other places. (P24, Site B)

## 4. Discussion

The COVID‐19 pandemic exposed vulnerabilities in health service providers’ emergency and pandemic preparedness and emphasized the need for a continuous cycle of planning, training, and revision of emergency protocols to address the evolving threat and maintain continuity of services [1, 29]. Central to the preparedness response is health service resilience, and the prerequisites that should be embedded during periods of normalcy, to ensure an effective response during a time of crisis [21]. As the COVID‐19 pandemic evolved, situations unfolded that were unfamiliar to most HCWs working in acute care, including the need to operationalize and sustain pandemic response plans over prolonged time periods. The study findings highlight the perceived lack of clarity around the components and processes required for a streamlined organizational pandemic response [30], particularly through the domains of governance, leadership, communication, and workforce preparedness. This resulted in the creation of department‐level systems and processes that contributed to an organizational response that was seen as chaotic and lacking cohesion. This finding is consistent with previous reports highlighting gaps in Australian pandemic response plans at both national and state levels [1].

Hospital governance and the ability to respond and prepare health service activity is an essential component of emergency and disaster preparedness and planning that include infectious disease outbreaks and pandemics [31–33]. Within the analytical framework, governance and leadership are fundamental building blocks of a resilient health system [21], which provide frontline HCWs with an effective governance model that provides the health system with leadership, strategy, policy, and accountability, while facilitating trust in the workforce [32]. At the study site, confusion and frustration were reported by HCW relating to inadequate training and support and a lack of involvement in pandemic planning, highlighting that the system‐level requirements for a coordinated pandemic response were lacking [18, 21]. Engaging with frontline HCWs in emergency response planning ensures the development of processes and procedures that are feasible and tailored to the clinical setting, while also developing familiarity with the required processes that will facilitate faster adaptation in the event of an emergency [31].

The study findings also highlight that before the pandemic there was a lack of embedded systems and structures to support frontline HCW well‐being, which is a critical requirement for organizational resilience and sustainability during a period of prolonged crisis [4, 21, 34]. Participants reported feeling overwhelmed by increased exposure risks and psychological distress associated with fear and risks to their health and that of their family members. In line with previous studies, participants from site B, which emerged as a COVID‐19 epicenter, reported increased levels of fatigue and exhaustion that was attributed to extended working hours and prolonged use of PPE [4, 23]. However, the impact of the pandemic was felt throughout the health service, as rapid changes to systems and structures unfolded, leading to role ambiguities and communication challenges associated with rapidly evolving information and the difficulties in disseminating fast‐paced updates to a diverse workforce, illustrating how pressure on systems and structures influences the organization’s capacity for resilience [4, 14].

While frontline HCWs are responsible for executing the emergency response, they require support from the governance structure behind them, to ensure the direction is clear, priority issues are understood, and that the response is cohesive. The analytical framework highlights how effective response strategies depend on the governance structure and strategies that influence decision‐making capability and communication pathways [21]. During the pandemic, health services had to rapidly adjust their existing governance structures to manage the emergent crisis while maintaining existing service delivery. The shift to a command‐and‐control strategy to manage the crisis aimed to centralize decision‐making to enable rapid responses, clear communication, streamline operations and resource allocation, and ensure consistency [35, 36]. This approach was unfamiliar to many HCWs, and the changes to everyday processes and local managers’ decision‐making autonomy left many HCWs feeling disconnected from the organization’s executive leadership team [37].

Emergency response planning and disaster training exercises were identified by participants as historically lacking but as potentially beneficial in highlighting deficits in organization‐wide emergency response systems [1]. Emergency preparedness exercises, including tabletop planning exercises and operational drills, have been identified as an effective strategy for assessing organizational capabilities to respond to a large‐scale health emergency and provides the opportunity to strengthen organizational resilience through preparedness of the health system’s foundational building blocks [21, 38]. The benefit of running simulated emergency exercises for large‐scale events is that these can enhance healthcare system resilience through improving protocols, as well as embedding governance structures such as command and control that can take effect during an emergency [39]. Simulated exercises provide frontline HCWs with a more comprehensive understanding of the strategies that need to be activated in a large‐scale emergency [40] and provide opportunities to address identified deficits [29, 40–42]. Emergency response exercises should therefore incorporate pandemic planning and recovery activities that engage organizational leaders and HCWs at local levels, to facilitate improved coordination and outcomes [18].

Organizational communication strategies were identified as being suboptimal, resulting in inconsistent and ineffective implementation of the pandemic response plan. Within the analytical framework, effective communication strategies are central to both preparedness and response capacity [21]. The impact of this undermined staff confidence, while rapidly changing government directives and resulting in an oversaturation of information created confusion [14, 43]. Communication has been identified to be most valuable to the workforce when it forms part of a centralized and coordinated response [23, 44, 45]. Communication modalities need to be multifaceted, as many clinical staff do not have the means to access emails at multiple times throughout the day [14]. Billings et al. [23]identified that signs and posters provided by leaders and communicated through a cascading approach were accessible and valued by the frontline staff. A comparative study of the leadership response to the pandemic, undertaken at the same health service, identified similar findings of challenges with communication related to the frequency of changing guidelines as the pandemic evolved, and the difficulties in communicating this to frontline HCWs [14]. The leadership group identified the communication strategy and employed a wide range of platforms to reach a wide audience [14]; however, this was not reported by the frontline HCWs who developed independent local strategies to reach their teams. These findings highlight the importance of a robust multifaceted communication strategy to reach all members of the HCW team, while remaining agile and responsive.

In line with previous studies, inadequate staff preparation heightened fears and reduced the HCWs’ capacity to respond effectively [14, 46]. Fear has a powerful influence on people, impacting workforce resilience, and should not be underestimated within a workforce setting [17]. The findings of fear amongst HCWs within this study were not unique and are consistent with studies of other respiratory pandemics [47, 48]. The psychological impact of COVID‐19 on frontline HCWs, including expressions of fear of the unknown, fear of becoming infected, and spreading infection to family members, was evident [47, 49]. Variations in information, equipment, and supplies, including changing PPE and accessibility to critical resources, also contributed to workforce fear and panic [17, 50, 51]. Organization and system‐wide strategies to address fear include targeted education, organization‐wide communication systems to reduce disparities in messaging, and understanding and encouraging and providing mental health support and initiatives is warranted [17].

While the psychological impact of the pandemic on frontline staff was not the focus of this study, the lack of organizational well‐being support was raised by participants, who identified frontline colleagues and peers as their main source of well‐being support [23, 46]. Stressors within the workplace as a result of increasing workloads, high patient acuity, and staff shortages have been previously reported [47]. Factors that increase workforce stress can result in a decrease in the quality of care patients receive and can extend to workforce withdrawal and staffing deficits with substantial impact on the staff who remain [4, 52]. The outcomes of this study identified two important considerations for institutional support to frontline staff to be effective: it needed to be accessible, coherent, and consistently communicated, and managers would benefit from training in psychological awareness and mental health support systems [46].

A lack of support and available counseling services was noted by participants, although these services, when available, are acknowledged as beneficial [50, 53]. While initiatives, in the form of food and well‐being tokens, were offered to frontline HCWs throughout the pandemic, evidence suggests that changes in models of healthcare delivery to include the provision of sustained structural supports for HCWs are better received [47]. Findings of this study suggest the need for greater social and psychological supports from the health service and colleagues, as well as the dissemination of clear information and guidance related to the disease, as a fundamental part of an emergency response [54]. Embedding well‐being support systems into pandemic response plans is an essential resilience prerequisite, whereby organizational leaders consider issues impacting the workforce such as: fatigue from extended use of PPE and the need for provision of extra breaks, increased locations for breaks, and hydration and food to alleviate stressors associated with work disruptions. Structured organizational responses that integrate governance, communication, and workforce support have the potential to strengthen health system resilience and sustainability during a crisis, and to improve the frontline HCWs’ experiences and confidence in the workplace response.

## 5. Limitations

This study, undertaken in 2023, asked participants to reflect on their experiences during the pandemic response from 2020 to 2023, which may be potentially impacted by the participants’ recall and retrospective interpretation of events. However, being removed from the immediate pressure of the crisis period enabled participants to provide more thoughtful and nuanced insights into their experiences over the 3 years. The large sample size and inclusion of participants from across the organization provide reassurance that the findings are representative of frontline HCWs’ experiences. Notably, the variation in context between the two hospital sites, with site B being at the epicenter of a large community outbreak, allowed the researchers to explore diverse perspectives and experiences. Given the structure of this health service, the results and experiences can be transferable to similar contexts. This study was conducted at one large organization in Australia; therefore, findings may not be transferable internationally.

## 6. Implications for Clinical Practice

A robust and resilient health service must respond to a crisis in a way that facilitates the continued provision of services to the population but also provides support to the frontline HCWs. The establishment of resilience prerequisites during normal time periods, which are embedded into the organization’s governance and communication models that become familiar to HCWs, is critical to the preparedness of an organization during an emergency. As the largest group of healthcare professionals, nurses formed a critical component of the pandemic response, and their frontline roles during the pandemic provide opportunities to understand the acute experience and reflect on the health service resilience prerequisites that can be strengthened to improve the pandemic response. This offers significant value to the formation of policy and future emergency response plans to provide more resilient healthcare delivery in the future [55]. The findings of this study indicate that there are opportunities to strengthen the health service preparedness and response capability across several of the analytical resilience framework domains [21]. Through the governance and leadership domain, there is opportunity to strengthen HCW preparedness by implementing mandatory, regular, emergency response training and simulation exercises. Creating and implementing leadership development models that include a focus on decision‐making, crisis management, and response strategies will further improve the frontline HCWs’ ability to lead in both normal and crisis periods and provide a coordinated leadership style across the organization. Embedding this as a resilience prerequisite provides several benefits, including clarifying roles, command and control structures, and communication channels, and it allows pandemic plans to be tested before a crisis impacts the health service. Improving and embedding a structured, centralized communication system will strengthen the information and service delivery domains and ensure communication structures are in place and have the capacity to provide a coordinated system‐wide response. Within the domain of human resources, improving and embedding well‐being and psychological support services for HCWs, which include proactive well‐being initiatives, will enable HCWs to process stressful and traumatic experiences, develop coping strategies, and reduce the risk of stress and burnout. Improving this domain helps to sustain workforce capacity and retain skilled HCWs within the health service, which further enables the health service to adapt and recover from a crisis, and contributes to sustaining safe and effective patient care.

Following completion of this study with frontline workers and separate interviews with organizational leaders [14], the key findings were reported back to the organizational COVID‐19 working group and informed an update of the organization’s pandemic response plan. The vulnerabilities and psychological impact of the pandemic on the nursing workforce not only has implications for improvements in patient outcomes but also provides strategies to invest in system‐level structures and workforce supports that will improve culture and build a sustainable nursing workforce.

## 7. Conclusion

During the COVID‐19 pandemic, acute health services were required to respond rapidly to emerging issues while maintaining the delivery of essential services. The insights and experiences of the frontline HCWs identify opportunities for improvement in the organizational response and demonstrate that pressure faced during the crisis period exposed gaps in the preparedness and resilience of the health service, in relation to the governance structure, workforce readiness, and communication systems. These findings highlight the importance of a strategic, coordinated, system‐wide response from leadership that involves frontline staff in the emergency planning and decision‐making processes. Doing so offers an opportunity to develop effective, sustainable, and resilient healthcare systems. Embedding staff well‐being initiatives into emergency response plans is a core component of resilience, promoting a confident and empowered workforce through communication strategies that are consistent and transparent, combined with robust education programs that embed infection prevention and control, will strengthen the workforce, and ultimately improve patient care provision and services.

**Nomenclature**
ANUMs: Associate nurse unit managers
COVID‐19: Coronavirus disease
DHHS: Department of health and human services
ED: Emergency department
HCW: Healthcare worker
HREC: Human research ethics committee
ICU: Intensive care unit
IPC: Infection prevention and control
NUMs: Nurse unit managers
PPE: Personal protective equipment

## Author Contributions

Karen Mckenna, Ana Hutchinson, Bernice Redley, and Stéphane Bouchoucha contributed to the conception and design of the study. Karen Mckenna, Ana Hutchinson, and Stéphane Bouchoucha analyzed and interpreted the data, and drafted and critically revised the paper.

## Funding

No funding was received for this study. Open access publishing facilitated by Deakin University, as part of the Wiley ‐ Deakin University agreement via the Council of Australasian University Librarians.

## Disclosure

All authors approved the final manuscript.

## Ethics Statement

Ethics approvals for this protocol were obtained from the organization’s Human Research Ethics Committee (HREC) and the University Ethics Committee. All methods were carried out in accordance with relevant guidelines and regulations. Informed consent was obtained from all participants in this research.

## Consent

Please see Ethics Statement.

## Conflicts of Interest

The authors declare no conflicts of interest.

## Data Availability

The datasets generated and analyzed during the current study are not publicly available due to the confidential nature of the participant data but are available from the corresponding author on reasonable request.

## References

[1] Bush M. , Bouchoucha S. L. , Hutchinson A. , and Bennett C. M. , Forecasting Pandemic Quarantine in New Zealand and Australia: A Scoping Review of Quarantine Characteristics and Capabilities Within Preparedness Plans and Pandemic Exercise Reports from 2002 to 2019, Journal of Infection and Public Health. (2023) 16, no. 12, 2017–2025, 10.1016/j.jiph.2023.10.017. 37890225

[2] Honda A. , dAOS R. , Valéry R. , Kate Z. , and Gautier L. , Attributes and Organizational Factors that Enabled Innovation in Health Care Service Delivery During the COVID-19 Pandemic – Case Studies from Brazil, Canada and Japan, Health Systems & Reform. (2023) 9, no. 2, 10.1080/23288604.2023.2176022. 37023218

[3] Holton S. , Rasmussen B. , Crowe S. et al., Worsening Psychological Wellbeing of Australian Hospital Clinical Staff During Three Waves of the Coronavirus (COVID-19) Pandemic, Australian Health Review. (2023) 47, no. 6, 641–651, 10.1071/ah23120. 37844618

[4] Dempster P. , Hutchinson A. , Oldland E. , and Bouchoucha S. , Impact of the COVID-19 Pandemic on Emergency Department Team Dynamics and Workforce Sustainability in Australia. A Qualitative Study, International Emergency Nursing. (2023) 71, 10.1016/j.ienj.2023.101378. 37918279

[5] Dempster P. , Hutchinson A. , Oldland E. , and Bouchoucha S. L. , Australian Emergency Nurses’ Experiences of Working with Personal Protective Equipment During the COVID-19 Pandemic. A Qualitative Study, Australas Emerg Care. (2024) 27, no. 1, 63–70, 10.1016/j.auec.2023.08.003. 37679286

[6] Al Asfoor D. , Tabche C. , Al-Zadjali M. , Mataria A. , Saikat S. , and Rawaf S. , Concept Analysis of Health System Resilience, Health Research Policy and Systems. (2024) 22, no. 1, 10.1186/s12961-024-01114-w. PMC10996206 38576011

[7] Stobart A. and Duckett S. , Australia’s Response to COVID-19. Health Economics, Policy and Law. (2022) 17, no. 1, 95–106, 10.1017/s1744133121000244. PMC8365101 34311803

[8] Department of the Prime Minister and Cabinet , COVID-19 Response Inquiry Summary Lessons for the next Crisis, 2024, Commonwealth of Australia.

[9] Carter D. , The Use of Coercive Public Health and Human Biosecurity Law in Australia: an Empirical Analysis, The University of New South Wales Law Journal. (2020) 43, no. 1, 10.53637/KVCB1591.

[10] Zhang L. , Tao Y. , Zhuang G. , and Fairley C. K. , Characteristics Analysis and Implications on the COVID-19 Reopening of Victoria, Australia, Innovation (Camb). (2020) 1, no. 3, 10.1016/j.xinn.2020.100049. PMC7527351 33020751

[11] Trauer J. M. , Lydeamore M. J. , Dalton G. W. et al., Understanding How Victoria, Australia Gained Control of Its Second COVID-19 Wave, Nature Communications. (2021) 12, no. 1, 10.1038/s41467-021-26558-4. PMC8560916 34725323

[12] Victorian Department of Health , Victorian Coronavirus (COVID-19) Data [Wensite], 2021, Victorian Department of Health, https://www.dhhs.vic.gov.au/victorian-coronavirus-covid-19-data.

[13] Victorian Government , Po V. , Victoria’s Health System Preparing for COVID-19 Pandemic, 2020, Victorian Department of Health.

[14] McKenna K. , Bouchoucha S. , Redley B. , and Hutchinson A. , Building the Plane While Flying it Reflections on Pandemic Preparedness and Response; an Organisational Case Study, BMC Health Services Research. (2023) 23, no. 1, 10.1186/s12913-023-09874-x. PMC10472588 37658384

[15] Wiig S. and O’Hara J. K. , Resilient and Responsive Healthcare Services and Systems: Challenges and Opportunities in a Changing World, BMC Health Services Research. (2021) 21, no. 1, 10.1186/s12913-021-07087-8. PMC8487709 34602063

[16] Troisi A. , Nanni R. C. , Riconi A. , Carola V. , and Di Cave D. , Fear of COVID-19 Among Healthcare Workers: the Role of Neuroticism and Fearful Attachment, Journal of Clinical Medicine. (2021) 10, no. 19, 10.3390/jcm10194358. PMC8509269 34640375

[17] Cawcutt K. A. , Starlin R. , and Rupp M. E. , Fighting Fear in Healthcare Workers During the COVID-19 Pandemic, Infect Control Hospital Epidemiology. (2020) 41, no. 10, 1192–1193, 10.1017/ice.2020.315. PMC7338429 32580790

[18] Orhierhor M. , Pringle W. , Halperin D. , Parsons J. , Halperin S. A. , and Bettinger J. A. , Lessons Learned from the Experiences and Perspectives of Frontline Healthcare Workers on the COVID-19 Response: a Qualitative Descriptive Study, BMC Health Services Research. (2023) 23, no. 1, 10.1186/s12913-023-10062-0. PMC10559616 37805603

[19] Brune C. , Agerholm J. , and Liljas A. , Medical Doctors’ Perceptions of the Media Coverage During the Covid-19 Pandemic: a Case Study in Stockholm, Health Services Insights. (2023) 16, 10.1177/11786329231222168. PMC10752069 38152291

[20] Weishaar H. , Evans M. , Chemali S. et al., The Importance of Organisational Culture for Health System Resilience: a Qualitative Analysis of Factors that Supported Health Care Workers in Germany During the COVID-19 Pandemic, PLoS One. (2025) 20, no. 1, 10.1371/journal.pone.0317231. PMC11731743 39808651

[21] Reiss M. , Kraus M. , Riedel M. , and Czypionka T. , What Makes Health Systems Resilient? an Analytical Framework Drawing on European Learnings from the COVID-19 Pandemic Based on a Multitiered Approach, BMJ Public Health. (2024) 2, no. 1, 10.1136/bmjph-2023-000378. PMC11812772 40018222

[22] Alhouri A. , Abu Shokor M. , Marwa K. et al., COVID-19 and Its Impact on Healthcare Workers: Understanding Stigma, Stress, and Quality of Life, Cureus. (2023) 15, no. 4, 10.7759/cureus.37846. PMC10198658 37214008

[23] Billings J. , Ching B. C. F. , Gkofa V. , Greene T. , and Bloomfield M. , Experiences of Frontline Healthcare Workers and Their Views About Support During COVID-19 and Previous Pandemics: a Systematic Review and Qualitative meta-synthesis, BMC Health Services Research. (2021) 21, no. 1, 10.1186/s12913-021-06917-z. PMC8419805 34488733

[24] Smallwood N. , Karimi L. , Bismark M. et al., High Levels of Psychosocial Distress Among Australian Frontline Healthcare Workers During the COVID-19 Pandemic: a cross-sectional Survey, Genitics Psychiatary. (2021) 34, no. 5, 10.1136/gpsych-2021-100577. PMC8423519 34514332

[25] Werner R. M. and Glied S. A. , Covid-Induced Changes in Health Care Delivery-Can They Last?, New England Journal of Medicine. (2021) 385, no. 10, 868–870, 10.1056/NEJMp2110679. 34449184 PMC9623875

[26] McCallum J. and Howes D. , Defining Exploratory-Descriptive Qualitative (EDQ) Research and Considering Its Application to Healthcare2018.

[27] Doyle L. , McCabe C. , Keogh B. , Brady A. , and McCann M. , An Overview of the Qualitative Descriptive Design Within Nursing Research, Journal of Research in Nursing. (2020) 25, no. 5, 443–455, 10.1177/1744987119880234. 34394658 PMC7932381

[28] Braun V. and Clarke V. , Maher A. , Thematic Analysis: A Practical Guide, 2022, SAGE Publications Ltd, London.

[29] Herstein J. J. , Schwedhelm M. M. , Vasa A. , Biddinger P. D. , and Hewlett A. L. , Emergency Preparedness: what is the Future?, Antimicrob Steward Healthc Epidemiol. (2021) 1, no. 1, 10.1017/ash.2021.190. PMC9495548 36168490

[30] Sehgal N. J. and Milton D. K. , Applying the Hierarchy of Controls: what Occupational Safety Can Teach us About Safely Navigating the next Phase of the Global COVID-19 Pandemic, Frontiers in Public Health. (2021) 9, 10.3389/fpubh.2021.747894. PMC8602064 34805071

[31] Seyedin H. , Moslehi S. , Sakhaei F. , and Dowlati M. , Developing a Hospital Preparedness Checklist to Assess the Ability to Respond to the COVID-19 Pandemic, Eastern Mediterranean Health Journal. (2021) 27, no. 2, 131–141, 10.26719/2021.27.2.131. 33665797

[32] Phillips G. , Kendino M. , Brolan C. E. et al., Lessons from the Frontline: Leadership and Governance Experiences in the COVID-19 Pandemic Response Across the Pacific Region, Lancet Regional Health. Western Pacific. (2022) 25, 10.1016/j.lanwpc.2022.100518. PMC9259208 35818573

[33] Edelman A. , Allen T. , Devine S. et al., Hospitals Respond to Demand. Public Health Needs to Respond to Risk: Health System Lessons from a Case Study of Northern Queensland’s COVID-19 Surveillance and Response, BMC Health Services Research. (2024) 24, no. 1, 10.1186/s12913-023-10502-x. PMC10797896 38238735

[34] Sundararajan K. , Bi P. , Milazzo A. , Poole A. , Reddi B. , and Mahmood M. A. , Preparedness and Response to COVID-19 in a Quaternary Intensive Care Unit in Australia: Perspectives and Insights from Frontline Critical Care Clinicians, BMJ Open. (2022) 12, no. 2, 10.1136/bmjopen-2021-051982. PMC8819546 35121600

[35] Pettersson J. , Prytz E. , Friberg M. et al., Decision Making in a Strategic Medical Command and Control Team During the Covid-19 Pandemic: A Case Study, Disaster Medicine and Public Health Preparedness. (2024) 18, 10.1017/dmp.2024.95. 39291321

[36] Farcas A. , Ko J. , Chan J. , Malik S. , Nono L. , and Chiampas G. , Use of Incident Command System for Disaster Preparedness: a Model for an Emergency Department COVID-19 Response, Disaster Medicine and Public Health Preparedness. (2021) 15, no. 3, e31–e36, 10.1017/dmp.2020.210. 32576330 PMC7371845

[37] Hutchings S. D. , Perry J. , Mills A. , Bartley F. , Bartley M. , and Park C. L. , Command, Control and Communication (C(3)) During the COVID-19 Pandemic; Adapting a Military Framework to Crisis Response in a Tertiary UK Critical Care Centre, Journal of the Intensive Care Society. (2022) 23, no. 2, 162–169, 10.1177/1751143720982191. 35615232 PMC9125435

[38] Skryabina E. , Reedy G. , Amlôt R. , Jaye P. , and Riley P. , What is the Value of Health Emergency Preparedness Exercises? A Scoping Review Study, International Journal of Disaster Risk Reduction. (2017) 21, 274–283, 10.1016/j.ijdrr.2016.12.010.

[39] Abualenain J. , Alhajaji R. , and Kamel Alsulimani L. , A Systematic Review of the Efficacy of Full-Scale Simulation Exercises in Enhancing Hospital Disaster Preparedness, The Journal of Medicine, Law & Public Health. (2024) 4, no. 3, 419–446, 10.52609/jmlph.v4i3.133.

[40] Zhang D. , Fu M. , Zhang J. et al., Evaluating Whether Nonimmersion Virtual Reality Simulation Training Improves Nursing Competency in Isolation Wards: Randomized Controlled Trial, Journal of Medical Internet Research. (2025) 27, 10.2196/63131. PMC11786140 39819587

[41] Al-Elq A. H. , Simulation-Based Medical Teaching and Learning, J Family Community Med. (2010) 17, no. 1, 35–40, 10.4103/1319-1683.68787. 22022669 PMC3195067

[42] Zucco L. , Chen M. J. , Levy N. et al., Just-In-Time in Situ Simulation Training as a Preparedness Measure for the Perioperative Care of COVID-19 Patients, Simulation in Healthcare. (2023) 18, no. 2, 90–99, 10.1097/sih.0000000000000635. 35148284 PMC10081926

[43] McGuinness S. L. , Josphin J. , Eades O. et al., Organizational Responses to the COVID-19 Pandemic in Victoria, Australia: a Qualitative Study Across Four Healthcare Settings, Frontiers in Public Health. (2022) 10, 10.3389/fpubh.2022.965664. PMC9557753 36249244

[44] Sonis J. D. , Black L. , Baugh J. et al., Leveraging Existing Quality Improvement Communication Strategies During the COVID-19 Crisis, The American Journal of Emergency Medicine. (2020) 38, no. 7, 1523–1524, 10.1016/j.ajem.2020.04.021. 32312576 PMC7151532

[45] McBeth B. , Karanas Y. , Nguyen P. , Kurani S. , and Bhimani M. , Improving Communication Between Hospital Administrations and Healthcare Providers During COVID-19: Experience from a Large Public Hospital System in Northern California, Journal of Communication in Healthcare. (2021) 14, no. 4, 274–282, 10.1080/17538068.2021.1975250.

[46] Moyo I. , Mavhandu-Mudzusi A. H. , and Haruzivishe C. , Frontline Healthcare Workers’ Experiences of Providing Care During the COVID-19 Pandemic at a COVID-19 Centre in Bulawayo, Zimbabwe: a Phenomenological Study, Curationis. (2022) 45, no. 1, e1–e11, 10.4102/curationis.v45i1.2292. PMC9257684 35792610

[47] Bouchoucha S. L. and Hutchinson A. , Using Terror Management Theory to Understand Health Workers Burnout in Response to the COVID-19 Pandemic, Journal of Stress, Trauma, Anxiety and Resillence. (2022) 1, no. 2, 40–47, 10.55319/js.v1i2.18.

[48] Martínez-Jabares S. , López-Alonso A. I. , Calvo-Ayuso N. , Charneco-Salguero G. , Quiñones-Pérez M. , and Martínez-Fernández M. C. , Fear of Death, Emotional Intelligence and Resilience Among Healthcare Staff During COVID-19: a Correlative Study, Journal of Nursing Management. (2025) 2025, no. 1, 10.1155/jonm/7872841. PMC12074846 40365505

[49] Zhang L. , Flike K. L. , Gakumo C. A. , Shi L. , Leveille S. G. , and Thompson L. S. , Frontline Healthcare Workers’ Mental Distress, Top Concerns, and Assessment on Hierarchy of Controls in Response to COVID-19: a cross-sectional Survey Study, Human Resources for Health. (2021) 19, no. 1, 10.1186/s12960-021-00661-5. PMC8474834 34565407

[50] Ayton D. , Soh S. E. , Berkovic D. et al., Experiences of Personal Protective Equipment by Australian Healthcare Workers During the COVID-19 Pandemic, 2020: a cross-sectional Study, PLoS One. (2022) 17, no. 6, 10.1371/journal.pone.0269484. PMC9173633 35671287

[51] Hill M. , Smith E. , and Mills B. , Work-Based Concerns of Australian Frontline Healthcare Workers During the First Wave of the COVID-19 Pandemic, Australia NZ Journal Public Health. (2022) 46, no. 1, 25–31, 10.1111/1753-6405.13188. PMC9968589 34897889

[52] Cutler D. M. , Health System Change in the Wake of COVID-19, JAMA Health Forum. (2023) 4, no. 10, e234355–e, 10.1001/jamahealthforum.2023.4355. 37856097

[53] Palinkas L. A. , Whiteside L. , Nehra D. et al., Rapid Ethnographic Assessment of the COVID-19 Pandemic April 2020 ‘Surge’ and Its Impact on Service Delivery in an Acute Care Medical Emergency Department and Trauma Center, BMJ Open. (2020) 10, no. 10, 10.1136/bmjopen-2020-041772. PMC7577068 33082198

[54] Cabarkapa S. , Nadjidai S. E. , Murgier J. , and Ng C. H. , The Psychological Impact of COVID-19 and Other Viral Epidemics on Frontline Healthcare Workers and Ways to Address it: a Rapid Systematic Review, Brain Behaviour Immunology Health. (2020) 8, 10.1016/j.bbih.2020.100144. PMC7494453 32959031

[55] Reynolds N. R. , Baker D. , D’Aoust R. et al., COVID-19: Implications for Nursing and Health Care in the United States, Journal of Nursing Scholarship. (2023) 55, no. 1, 187–201, 10.1111/jnu.12853. 36583656 PMC9847252

